# Does AI Advertising Persuade or Scaffold? Consumer Cognitive Pathways in AI-Mediated Experiential Consumption

**DOI:** 10.3390/bs16081325

**Published:** 2026-08-03

**Authors:** Jung-eun Bae

**Affiliations:** Department of Business Administration, Pusan National University, Busan 46241, Republic of Korea; jeong-eun@snu.ac.kr; Tel.: +82-10-8786-0172

**Keywords:** AI-targeted advertising, cognitive scaffolding, consumer value construction, trust, purchase intention, experiential consumption, AI utilization, consumer decision-making

## Abstract

AI-targeted advertising has become central to consumer-facing marketing, yet it does not directly persuade consumers to purchase experiential products. This study proposes a cognitive scaffolding framework, arguing that AI advertising supports consumers’ autonomous value construction rather than directly generating purchase motivation. Using SEM with 412 Korean consumers in cultural experience tourism, the results show that AI advertising has no direct association with purchase intention. Instead, its association operates sequentially through perceived value and trust. Moreover, the value–purchase intention relationship is stronger among consumers with higher AI utilization. Complementary fsQCA shows that no single condition—including advertising effectiveness—is individually necessary for high purchase intention. A configurational analysis identifies a dominant sufficient path in which perceived value combines with AI utilization and does not require advertising effectiveness, alongside two secondary paths in which advertising effectiveness operates only in conjunction with other conditions. This pattern is consistent with a reconceptualization of AI advertising as one of several substitutable cognitive scaffolding inputs, interpretable as supporting rather than driving consumers’ autonomous value construction—a claim advanced as specific to experiential consumption contexts rather than as a general account of AI-mediated trust formation.

## 1. Introduction

AI-targeted advertising has fundamentally transformed how consumers discover and evaluate products, with algorithmic recommendation systems now mediating purchase decisions across virtually every consumption domain (Huang & Rust, 2021; Jain et al., 2024; Mogaji, 2024). Yet the consumer psychology underlying these AI-mediated decisions remains insufficiently understood, particularly for experiential products whose value resists the parametric logic of recommendation algorithms. A growing body of empirical evidence calls this assumption into question.

Cicek et al. (2024) demonstrated that merely describing products as “AI-powered” significantly lowered purchase intention across multiple product categories, with diminished emotional trust identified as the mediating mechanism. Park and Son (2025) found that consumers prefer AI-generated recommendations for utilitarian travel goals (e.g., business trips) but not for hedonic ones (e.g., romantic getaways). Li et al.’s (2024) meta-analysis of 91 AI hospitality studies revealed that AI’s service outcomes are mediated through customers’ cognition and affect rather than through direct behavioral conversion. Collectively, these findings point to a shared conclusion: the persuasion model—in which advertising stimuli directly convert to purchase behavior—may not apply when AI recommends experiential hospitality products.

What remains unresolved, however, is a theoretical account of what AI advertising does accomplish when direct persuasion fails. Existing studies have documented the phenomenon—AI advertising is not straightforwardly persuasive for experiential products—but have not specified the cognitive mechanism through which it is nonetheless associated with behavioral outcomes. These gaps are not unique to tourism but reflect broader unresolved questions in AI consumer behavior research. Jain et al. (2024) identified the cognitive mechanisms of AI-mediated decision-making as a priority research area, while Trang (2025) raised concerns about consumer over-dependency on AI and its implications for decision autonomy. We address these calls by drawing on the concept of cognitive scaffolding (Vygotsky, 1978; Wood et al., 1976) to propose an alternative to the persuasion model.

Cognitive scaffolding, in its original educational psychology formulation, refers to temporary support structures that enable learners to accomplish tasks they could not perform independently, while the learner—not the support—remains the author of the outcome (Vygotsky, 1978; Wood et al., 1976). We develop the three properties that define this concept, and their application to AI advertising, in Section 2.

We propose that these properties map onto the dynamics of AI-targeted advertising for experiential hospitality products. When a consumer encounters an AI-personalized recommendation for a temple stay or hanok stay, the advertising is not directly associated with purchase motivation (as persuasion theory would predict) but instead provides informational structure—curated options, relevance signals, preference-matched features—upon which the consumer constructs their own value judgment. This value judgment is then associated with trust in the recommendation source, which subsequently is linked to purchase commitment. The scaffolding framework further predicts that this process is moderated by consumer capability: individuals with higher AI utilization—analogous to Vygotsky’s more competent learners—should convert scaffolded value judgments into behavioral action more efficiently than those with lower AI utilization.

This theoretical framework generates specific predictions that differ from those of the persuasion model. Persuasion theory predicts a significant direct association between advertising effectiveness and purchase intention—the more persuasive the AI advertising, the stronger the purchase motivation. The scaffolding framework predicts no direct association, with the advertising’s association operating entirely through sequential mediation (perceived value → trust → purchase intention) and moderated by the consumer’s AI utilization level at the value-to-action stage. These competing predictions can be empirically discriminated, making the choice between frameworks a matter of evidence rather than terminological preference.

Three specific gaps in the existing literature motivate the present investigation. First, while prior evidence reviewed above establishes that AI advertising does not straightforwardly persuade for experiential and hedonic products, no study has empirically specified the alternative cognitive pathway through which AI advertising is associated with purchase intention when direct persuasion fails.

Second, the AI consumer behavior literature has extensively examined trust as a determinant of consumer responses—Shi et al. (2021) modeled it as a primary mediator, Bilgihan et al. (2026) positioned it as a parallel mediator, and technology acceptance models treat it as a precondition (Gefen et al., 2003)—but the sequential ordering between value assessment and trust formation remains untested for experiential consumption contexts. This matters because the ordering determines whether marketing interventions should target trust directly or focus on demonstrating recommendation value.

Third, although research has documented that consumers differ in their AI responses (Chi et al., 2022; Seyfi et al., 2025), no study has identified where in the cognitive process these individual differences operate—at the evaluation stage, the trust stage, or the action stage.

This article empirically examines two questions: (1) whether AI-targeted advertising for cultural experience tourism is associated with consumers’ purchase intention directly (as the persuasion model predicts) or through sequential mediation by perceived value and trust (as the scaffolding framework predicts); and (2) how these pathways are moderated by consumers’ AI utilization levels. By doing so, this research aims to contribute to the AI consumer behavior literature by specifying the cognitive mechanism through which AI advertising is associated with experiential consumption decisions, by testing the sequential ordering of value and trust in AI-mediated contexts, and by identifying the cognitive locus at which consumer AI utilization moderates the advertising-to-purchase process. The remainder of this paper is organized as follows. Section 2 develops the theoretical background and hypotheses; Section 3 describes the method; Section 4 reports the SEM and fsQCA results; Section 5 discusses theoretical and managerial implications, limitations, and future research.

## 2. Theoretical Background and Hypothesis Development

### 2.1. Two Models of AI Advertising Effects on Consumer Behavior in Experiential Consumption

The persuasion model, grounded in traditional advertising theory (MacInnis & Jaworski, 1989; MacKenzie & Lutz, 1989), treats AI-targeted advertising as an enhanced form of persuasive communication. By matching messages to individual preferences through algorithmic analysis of consumer data, AI increases relevance and thereby directly strengthens purchase intention (Chatterjee et al., 2022; Hollebeek et al., 2024). This model has been productively applied to standardized products and search goods, where attribute comparison is the primary decision mechanism and algorithmic recommendations reduce information search costs (Adomavicius & Tuzhilin, 2005; Huang & Rust, 2021). Under the persuasion model, AI advertising should exhibit a significant direct association with purchase intention, with mediation through cognitive variables serving as an additional (but not exclusive) pathway. The cognitive scaffolding model, which we develop below, offers a fundamentally different prediction. Drawing on Vygotsky’s (1978) zone of proximal development and Wood et al.’s (1976) formalization of the scaffolding concept, this model proposes that AI advertising for experiential hospitality products functions not as persuasion but as evaluative support. The scaffolding framework is grounded in three theoretically derived properties. First, scaffolding provides structure without dictating conclusions—AI advertising curates options and signals relevance, but the consumer constructs the value judgment autonomously. Second, scaffolding enables performance that the individual cannot achieve alone—consumers cannot efficiently evaluate experiential options across a vast information space without algorithmic curation, but the curation does not substitute for their own evaluative judgment. Third, scaffolding effectiveness is contingent on the user’s capability—individuals with greater relevant competence utilize scaffolding more efficiently (Puntambekar & Hübscher, 2005). Having defined these three properties, we next clarify how cognitive scaffolding differs from related constructs, since its theoretical contribution depends on this distinction. Distinguishing cognitive scaffolding from adjacent constructs. Because several established constructs also describe how informational inputs shape consumer judgment, it is important to specify what cognitive scaffolding adds. Information support, recommendation usefulness, decision support, perceived diagnosticity, and personalization each characterize a property of the informational stimulus—its quantity, relevance, or fit. Cognitive scaffolding, by contrast, characterizes a relational process between the support and the consumer’s evolving competence. Crucially, a highly diagnostic or personalized recommendation can still operate through pure persuasion; what distinguishes scaffolding is not the richness of the information but its calibration to—and eventual dispensability for—a competent consumer. Constructs describing the stimulus alone therefore cannot capture the scaffolding dynamic, which is why an additional relational construct is warranted. Its distinguishing observable implication is asymmetric substitutability: a persuasive input is expected to contribute wherever it is present, whereas a scaffolding input should be dispensable precisely when the consumer already commands the resources it would otherwise supply. This prediction is directly testable and differentiates scaffolding from a generic indirect-effect account: if AI advertising functions as scaffolding, it should be absent from at least one sufficient path to high purchase intention among consumers who already possess strong value-construction resources. We specify the three scaffolding properties as the construct’s defining features rather than claiming to measure them directly, and we return to their operationalization in the Limitations. With this conceptual distinction established, the scaffolding model yields concrete, testable predictions about how AI advertising operates.

Under the scaffolding model, AI advertising should exhibit no significant direct association with purchase intention. Instead, its association should operate entirely through sequential mediation: advertising provides informational cues → consumers use these cues to construct perceived value → value recognition is associated with trust in the information source → trust is associated with purchase commitment. Furthermore, the value-to-action conversion should be moderated by consumers’ AI utilization level, with higher-utilization consumers converting more efficiently—paralleling the scaffolding prediction that more competent individuals benefit from scaffolding more effectively.

These competing predictions—direct association (persuasion) versus full mediation with capability-contingent moderation (scaffolding)—can be empirically discriminated using structural equation modeling. The S–O–R framework (Mehrabian & Russell, 1974) provides the structural architecture for this test, with advertising effectiveness as the stimulus (S), perceived value and trust as sequential organism processes (O), and purchase intention as the response (R).

Whether the persuasion or scaffolding model better accounts for the data has direct implications for how hospitality marketers should design, deploy, and evaluate AI advertising systems. If persuasion holds, optimization should target persuasive intensity. If scaffolding holds, optimization should target evaluative support quality.

Several recent findings support the plausibility of the scaffolding model for experiential contexts. As reviewed in the Introduction, evidence indicates that AI’s persuasive capacity is limited for hedonic and experiential goods and that its effects operate through cognition and affect rather than direct conversion (Cicek et al., 2024; Park & Son, 2025; Li et al., 2024). Two further findings sharpen this case. Chi et al. (2020) showed that AI acceptance in hospitality depends on the service encounter and the perceived role of human judgment. Seyfi et al. (2025) identified loss of autonomy as a barrier to AI adoption—a concern that scaffolding would mitigate by preserving consumer agency, but that persuasion would not.

Cultural experience tourism—temple stay, hanok stay, meditation programs, traditional cultural workshops—represents a strong test case for discriminating between the two models. These are prototypical experience goods (Nelson, 1970) whose quality can only be assessed through consumption, and whose value derives from subjective, embodied dimensions that resist algorithmic parameterization. If the scaffolding model is supported in this context, it would suggest that AI advertising’s function shifts from persuasion to evaluative support as product experientiality increases—a boundary condition with significant implications for hospitality marketing theory.

Following Pine and Gilmore’s (1998) experience economy framework, these products share core experiential characteristics: active participation, immersion, and personal transformation. These characteristics distinguish them from the passive consumption of standardized hospitality services and render their value resistant to algorithmic parameterization.

### 2.2. Advertising Effectiveness, Perceived Value, and Trust as Sequential Cognitive Processes

Within the S–O–R framework, advertising effectiveness constitutes the external stimulus. Following MacKenzie and Lutz (1989) and Ducoffe (1996), advertising effectiveness captures consumers’ overall evaluation of the informativeness, relevance, and persuasive quality of AI-targeted advertising for cultural experience tourism. Perceived value represents consumers’ overall assessment of expected benefits relative to sacrifices (Zeithaml, 1988). In experiential hospitality contexts, perceived value encompasses functional utility (information quality, search efficiency), hedonic anticipation (expected enjoyment, aesthetic pleasure), and symbolic meaning (cultural enrichment, self-development). We hypothesize that effective AI-targeted advertising enhances perceived value by providing personalized information that helps consumers identify experiences aligned with their preferences—serving as informational scaffolding that facilitates value recognition without dictating value conclusions.

**H1.**  *Advertising effectiveness is positively associated with perceived value.*

Trust reflects consumers’ confidence that the AI recommendation source is reliable, competent, and aligned with their interests (Morgan & Hunt, 1994; Gefen et al., 2003). Shi et al. (2021) distinguished cognitive trust (based on evidence of competence) from emotional trust (based on interpersonal warmth and care), finding that emotional trust exerts a stronger influence on AI delegation decisions. We argue that, in the context of AI-targeted advertising for experiential hospitality, trust formation encompasses both dimensions. Consumers assess whether the algorithm identifies genuinely valuable experiences (cognitive trust) and whether the recommendation appears to have their best interests in mind (emotional trust).

**H2.**  *Advertising effectiveness is positively associated with trust.*

### 2.3. Competing Predictions: Direct Association Versus Full Mediation

The persuasion and scaffolding models generate divergent predictions regarding the direct path from advertising effectiveness to purchase intention. Traditional advertising theory posits a direct stimulus-to-response pathway (MacInnis & Jaworski, 1989), and the persuasion model predicts that AI’s algorithmic personalization strengthens this pathway by increasing message relevance (Huang & Rust, 2021). The scaffolding model, by contrast, predicts that this direct pathway will be non-significant for experiential hospitality products: advertising provides informational structure for consumer-driven value construction, but is not directly associated with purchase motivation. To discriminate between these models, we test the direct association hypothesis:

**H3.**  *Advertising effectiveness is positively associated with purchase intention.*

If H3 is supported, the persuasion model is favored: AI advertising directly generates purchase motivation for cultural experience tourism. If H3 is not supported while the mediated pathways (H7–H9) are significant, the scaffolding model is favored: AI advertising functions as evaluative infrastructure rather than as a persuasive tool. This formulation treats H3 not as a hypothesis expected to succeed or fail, but as a diagnostic test between two competing theoretical frameworks.

### 2.4. Sequential Mediation: Value Construction and Trust Formation

The scaffolding framework predicts a specific sequential structure within the mediated pathway. In Vygotsky’s (1978) formulation, scaffolding operates by enabling the supported individual to first construct understanding and then develop confidence in that understanding. Translated to the AI advertising context, this sequence unfolds in two steps. Consumers first use AI-provided informational cues to construct perceived value, assessing whether the recommended experience offers genuine benefits. They then develop trust in the recommendation source based on the quality of that value assessment. This sequential ordering differs from models that treat trust as a precondition for engagement (Gefen et al., 2003) or as a parallel input alongside value (Dodds et al., 1991). Our rationale is that for experience goods characterized by high pre-purchase uncertainty, consumers engage in prospective value construction—mentally simulating the anticipated experience based on available informational cues (Tussyadiah et al., 2023). We therefore propose that, in this context, value construction tends to precede trust formation: consumers are unlikely to trust a recommendation system until they have some evidence that its recommendations identify genuinely valuable experiences. We advance this as a theoretical proposition to be tested rather than as a logical necessity, and we note that our cross-sectional design cannot confirm the temporal sequence directly. This proposition is consistent with Shi et al.’s (2021) finding that systematic cues exert stronger effects on AI adoption than heuristic cues, which points to deliberate value assessment—a systematic process—as a plausible anchor for subsequent trust formation. We acknowledge that an influential literature specifies the reverse ordering, treating trust as antecedent to value; Sirdeshmukh et al. (2002), for instance, position value as a mediator of the trust–loyalty relationship. That tradition, however, developed largely in contexts where quality is verifiable and the consumer’s task is to decide whether to rely on a provider. Experiential consumption differs fundamentally: because value cannot be verified before purchase, it must be actively constructed, so an initial value judgment can precede and inform trust in the recommending agent. Rather than assuming either ordering, we test both empirically (Section 4.4) and adopt a configurational approach (Section 4.6), precisely because two competing orderings cannot be adjudicated by a single structural specification.

**H4.**  *Perceived value is positively associated with trust.*

**H5.**  *Perceived value is positively associated with purchase intention.*

**H6.**  *Trust is positively associated with purchase intention.*

**H7.**  *Perceived value mediates the relationship between advertising effectiveness and purchase intention.*

**H8.**  *Trust mediates the relationship between advertising effectiveness and purchase intention.*

**H9.**  *Advertising effectiveness has an indirect association with purchase intention through the sequential mediation of perceived value and trust.*

### 2.5. Capability-Contingent Moderation: AI Utilization Level

A distinctive property of scaffolding, as theorized by Vygotsky (1978) and elaborated by Wood et al. (1976), is that its effectiveness varies with the learner’s competence. More capable individuals utilize scaffolding more efficiently—not because they perceive the scaffold differently, but because they translate the scaffolded understanding into independent action more readily. In the AI advertising context, this translates to a prediction about where AI utilization should moderate the advertising-to-purchase process.

We theorize that consumers with higher AI utilization—greater habitual experience with AI-based decision tools (Venkatesh et al., 2003; Compeau & Higgins, 1995)—possess greater fluency in interpreting and acting upon AI-generated recommendations. This fluency should strengthen the perceived value → purchase intention pathway, as high-utilization consumers more readily translate value assessments into purchase decisions with less deliberation. Crucially, the scaffolding framework predicts that this moderation operates at the value-to-action stage, not at the evaluation or trust stages: both high- and low-utilization consumers should be equally capable of recognizing value and forming trust from AI recommendations, but they should differ in the cognitive efficiency with which they convert these evaluations into behavioral commitment.

This prediction is consistent with Li et al.’s (2024) meta-analytic finding that AI’s service outcomes are mediated through cognition, while specifying the point at which individual cognitive differences become consequential. It also extends Seyfi et al.’s (2025) identification of psychological barriers to AI adoption by proposing a specific cognitive locus for these barriers: not at the perception or evaluation stage, but at the action-conversion stage.

**H10.**  *The association between perceived value and purchase intention differs by AI utilization level.*

**H11.**  *The association between advertising effectiveness and purchase intention differs by AI utilization level.*

**H12.**  *The association between trust and purchase intention differs by AI utilization level.*

## 3. Research Methodology

### 3.1. Research Model

**Figure 1:**
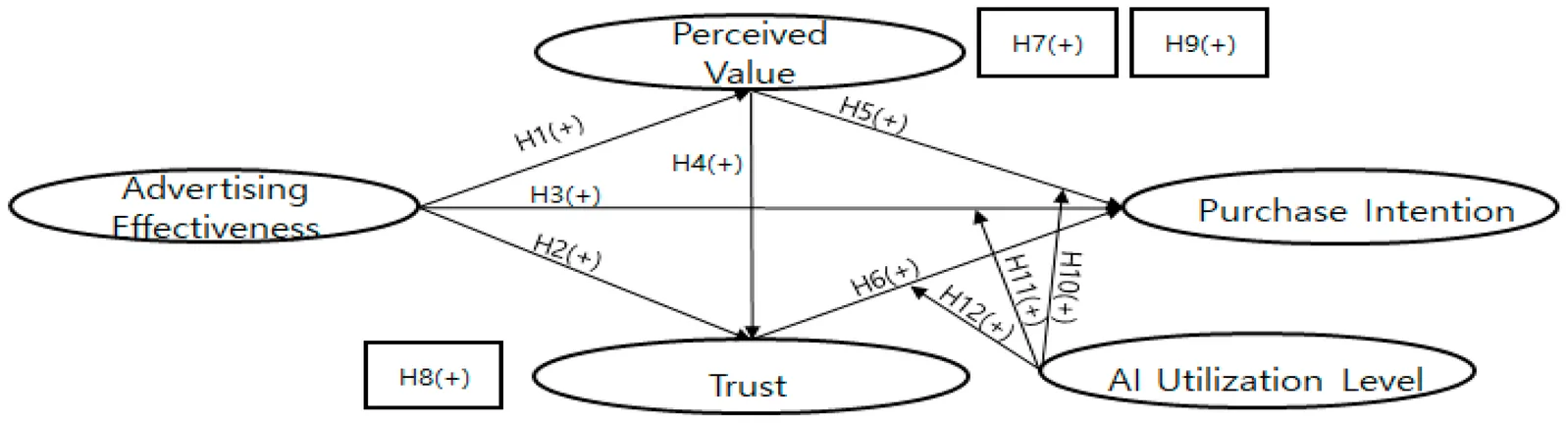
Research Model.

The research model is grounded in the S–O–R framework. AI-targeted advertising effectiveness functions as the external stimulus (S) activating internal cognitive evaluations—perceived value and trust (O)—which subsequently shape purchase intention (R). AI utilization level is incorporated as a moderator of the organism-to-response pathways. The proposed model is presented in Figure 1.

### 3.2. Operational Definitions

All constructs were measured using established scales with minimal contextual adaptation for cultural experience tourism.

Advertising effectiveness refers to consumers’ overall evaluation of AI-targeted cultural experience tourism advertising, including its informativeness, relevance, and persuasive quality (MacKenzie & Lutz, 1989; Ducoffe, 1996). Perceived value captures consumers’ subjective assessment of expected experiential benefits relative to anticipated costs (Zeithaml, 1988). Trust measures the extent to which consumers believe the AI recommendation source is reliable and capable of meeting expectations (Morgan & Hunt, 1994; Gefen et al., 2003). Purchase intention reflects consumers’ behavioral intention to book or participate in cultural experience tourism programs (Dodds et al., 1991; Ajzen, 1991). AI utilization level captures self-assessed experience with and habitual use of AI-based tools for information search and decision-making (Compeau & Higgins, 1995; Venkatesh et al., 2003). We interpret AI utilization as a proxy for consumer competence—the accumulated skill and fluency that shape how effectively a consumer can act on algorithmic support—rather than as familiarity, technological readiness, prior positive experience, or general attitude toward AI. This interpretation follows from the scaffolding logic, in which the relevant moderator is the learner’s current ability level. We nonetheless acknowledge in the Limitations that a single behavioral-frequency measure cannot fully disentangle competence from these adjacent constructs. Detailed measurement items are presented in Appendix A.

### 3.3. Sample and Data Collection

The target population consisted of Korean consumers who had recent experience searching for or engaging with cultural experience tourism products. Data were collected through an online survey distributed over a 14-day period from 23 February to 8 March 2026. A total of 427 responses were collected. After excluding 15 responses due to incomplete or inattentive answering patterns, 412 valid responses were retained for the final analysis.

To ensure respondent relevance, a multi-stage screening procedure was employed. First, respondents were asked whether they had searched for or booked cultural experience tourism products—such as temple stay, hanok stay, traditional cultural programs, meditation experiences, or traditional food experiences—within the past six months, or were currently planning such an experience. Respondents who reported no relevant experience or intention were excluded. This screening prioritizes construct relevance—ensuring that every respondent had genuine, conscious exposure to the AI-mediated advertising environment under study—over unrestricted representativeness, a trade-off appropriate to a study of psychological mechanisms rather than population prevalence.

Second, respondents were screened for exposure to AI- or algorithm-based personalized recommendation advertising during their cultural tourism information search on digital platforms. Only those who reported such exposure proceeded to the main survey.

Third, respondents were asked whether the advertisement or recommendation contained explicit cues indicating AI or personalization (e.g., “AI-recommended,” “personalized for you,” or “based on your interests”). Respondents who could not recognize or recall such cues were excluded, ensuring that participants had actual conscious experience with AI-mediated advertising environments. These specific cultural tourism offerings were selected because they represent officially designated and institutionally managed experiential programs actively promoted through AI-enabled digital platforms (e.g., VisitKorea, templestay.com), ensuring ecological validity in respondents’ exposure to AI-targeted advertising. The demographic characteristics of the final sample are reported in Table 1.

### 3.4. Validity and Reliability of Measurement Scales

An exploratory factor analysis (EFA) was conducted to assess the validity of the measurement scales. Principal component analysis with Varimax rotation was employed, and items with factor loadings below 0.50 were considered inadequate indicators of the underlying constructs (Hair et al., 1998). Scale reliability was evaluated using Cronbach’s alpha coefficients, with values above 0.60 regarded as acceptable (Nunnally, 1978).

As shown in Table 2, the Kaiser–Meyer–Olkin (KMO) measure of sampling adequacy was 0.923, exceeding the recommended threshold. Bartlett’s test of sphericity was also significant (*p* < 0.001), indicating that the data were suitable for factor analysis. Five factors with eigenvalues greater than 1.0 were extracted, and all items exhibited factor loadings above 0.50. Cronbach’s alpha values were 0.87 for advertising effectiveness, 0.94 for perceived value, 0.86 for trust, 0.88 for AI utilization level, and 0.90 for purchase intention, demonstrating satisfactory internal consistency and reliability across all constructs.

### 3.5. Data Analysis

The collected data were analyzed using SPSS 27.0 and AMOS 23.0. Exploratory factor analysis and Cronbach’s alpha coefficients were employed to assess the validity and reliability of the measurement scales, while descriptive statistics and Pearson correlation analysis were conducted to examine variable distributions and interrelationships. Confirmatory factor analysis (CFA) was subsequently performed to evaluate the convergent and discriminant validity of the measurement model.

To test the proposed hypotheses, structural equation modeling (SEM) was applied to estimate path coefficients and mediation effects. Model fit was assessed using the IFI, TLI, CFI, and RMSEA indices. Values of IFI, TLI, and CFI above 0.90 were considered indicative of acceptable fit (Bentler, 1990), and RMSEA values below 0.05 indicated good fit, values below 0.08 reasonable fit, and values below 0.10 mediocre but acceptable fit (Browne & Cudeck, 1992). In addition, multi-group analysis (MGA) was conducted to examine the moderating effects of AI utilization level. To complement the SEM analysis, fuzzy-set qualitative comparative analysis (fsQCA) was employed to examine configurational patterns leading to high purchase intention (Ragin, 2008). While SEM identifies the net effects of individual variables, fsQCA reveals how combinations of conditions jointly produce the outcome, capturing equifinality (multiple pathways to the same outcome), conjunctural causation (conditions working together), and asymmetry (conditions for the presence vs. absence of the outcome may differ) (Fiss, 2011). Analyses were performed using the QCA package (Dușa, 2019) in R. Calibration followed the direct method (Ragin, 2008). Because the scale-point anchors initially considered (2.0/3.0/4.0) produced severely skewed set memberships—construct means of 3.2–3.7 lie close to the 4.0 full-membership anchor, which inflates necessity consistency estimates (Schneider & Wagemann, 2012)—we adopted sample-based anchors at the 95th percentile (full membership), 50th percentile (crossover), and 5th percentile (full non-membership): AE (2.50/3.75/5.00), PV (2.18/3.33/5.00), TR (2.33/3.67/5.00), AIU (2.00/3.33/4.67), and PI (1.67/3.00/4.67). Because the composite scores are discrete, the crossover anchor was set marginally between adjacent observed values to avoid membership scores of exactly 0.50, ensuring that all 412 cases entered the truth table (Ragin, 2008). Necessity was assessed against the conventional 0.90 consistency benchmark. Truth table rows were retained with a frequency cutoff of 5 cases, a raw consistency threshold of 0.80, and a PRI consistency threshold of 0.65; the PRI criterion screens out rows exhibiting simultaneous subset relations with both the outcome and its negation despite high raw consistency (Schneider & Wagemann, 2012). The conservative (complex) solution is reported, as it makes no assumptions about unobserved logical remainders.

Given that this study aims to investigate whether the persuasive effects of AI-targeted advertising for experiential products operate indirectly through perceived value and trust rather than directly influencing purchase intention, SEM and MGA were deemed appropriate analytical approaches for simultaneously testing multiple mediation pathways and moderation effects. Because all constructs were measured from a single source at a single time point, we assessed common method bias using a confirmatory factor analysis approach that compares a single-factor model against the hypothesized multi-factor model (Podsakoff et al., 2003). Specifically, we contrasted a multi-factor model, in which each key variable was specified as a distinct latent construct, with a single-factor model in which all measurement items loaded on one common latent factor. The single-factor model exhibited poor fit, falling substantially below minimum acceptable thresholds (IFI = 0.675, TLI = 0.609, CFI = 0.674, RMSEA = 0.225). Moreover, the chi-square difference between the two models was statistically significant at the 0.05 level, confirming the superiority of the multi-factor model. Because the observed covariance structure is not attributable to a single underlying factor, common method bias is unlikely to pose a serious threat in this study. Nevertheless, given the single-source cross-sectional design, we acknowledge this constraint in the limitations.

## 4. Research Results

### 4.1. Descriptive Statistics and Correlation Analysis

Descriptive statistics were conducted to examine the distributional properties and normality of the key variables, including advertising effectiveness, perceived value, trust, AI utilization level, and purchase intention (Table 3). The skewness values ranged from −0.20 to 0.22, and kurtosis values ranged from −0.39 to 0.29. All variables satisfied the normality assumptions, as the absolute values of skewness were below 3 and kurtosis values were below 7, consistent with the criteria suggested by Kline (2005).

Pearson correlation analysis was subsequently conducted to examine the relationships among the key variables, and the results are presented in Table 4. All correlations were positive and statistically significant, with correlation coefficients ranging from 0.53 to 0.72.

### 4.2. Measurement Model Assessment

The measurement model yielded χ^2^(59) = 242.01 (*p* < 0.001), IFI = 0.956, TLI = 0.942, CFI = 0.956, and RMSEA = 0.087. The incremental indices exceeded the 0.90 benchmark, whereas the RMSEA falls within the mediocre-but-acceptable band under Browne and Cudeck’s (1992) criteria rather than the close-fit range—a level plausible given the model’s complexity relative to the sample size; larger samples or the inclusion of method factors may improve absolute fit in future work. The measurement model demonstrated an acceptable level of fit, with χ^2^(59) = 242.01 (*p* < 0.001), IFI = 0.956, TLI = 0.942, CFI = 0.956, and RMSEA = 0.087. Factor loadings, average variance extracted (AVE), and construct reliability (CR) for the measurement model are reported in Table 5. We note that the RMSEA of 0.087 falls in the mediocre-but-acceptable band under Browne and Cudeck’s (1992) criteria rather than the close-fit range. This level is plausible given the model’s complexity relative to the sample size; the residual misfit is concentrated in the strong covariation between advertising-effectiveness and trust indicators. The incremental indices (CFI = 0.956, TLI = 0.942) exceeded conventional thresholds, and future work with larger samples may improve absolute fit. Following the criteria proposed by Anderson and Gerbing (1988), convergent validity is supported when standardized factor loadings and AVE exceed 0.50 and CR exceeds 0.70. In this study, standardized factor loadings ranged from 0.72 to 0.94, AVE values from 0.62 to 0.84, and CR values from 0.87 to 0.94, indicating that all constructs satisfied the recommended thresholds and that convergent validity was adequately established.

Discriminant validity was assessed using the Fornell–Larcker criterion, which requires the square root of the average variance extracted (AVE) for each construct to exceed the absolute values of its correlations with other constructs (Fornell & Larcker, 1981). The inter-construct correlations and the square roots of AVE are reported in Table 6. The results indicate that the square root of AVE for each construct was greater than the corresponding inter-construct correlations, thereby confirming adequate discriminant validity. However, the latent correlation between advertising effectiveness and trust (0.77), while satisfying the Fornell–Larcker criterion, is high enough to warrant further examination. As a more conservative check, we computed the heterotrait–monotrait (HTMT) ratio of correlations (Henseler et al., 2015). All HTMT values fell below the conservative 0.85 threshold, with the highest—between advertising effectiveness and trust—at 0.829, supporting discriminant validity. Conceptually, the two constructs remain distinct: advertising effectiveness captures the consumer’s evaluation of the advertisement as informative and useful, whereas trust captures reliance on the AI source as a basis for action. Their empirical proximity is theoretically sensible—an effective, informative recommendation is a natural antecedent of trust—without implying redundancy. We nonetheless treat this proximity as a point requiring interpretive caution rather than a fully resolved issue, and we return to it in the Limitations.

### 4.3. Mediation Effect Analysis

**Figure 2:**
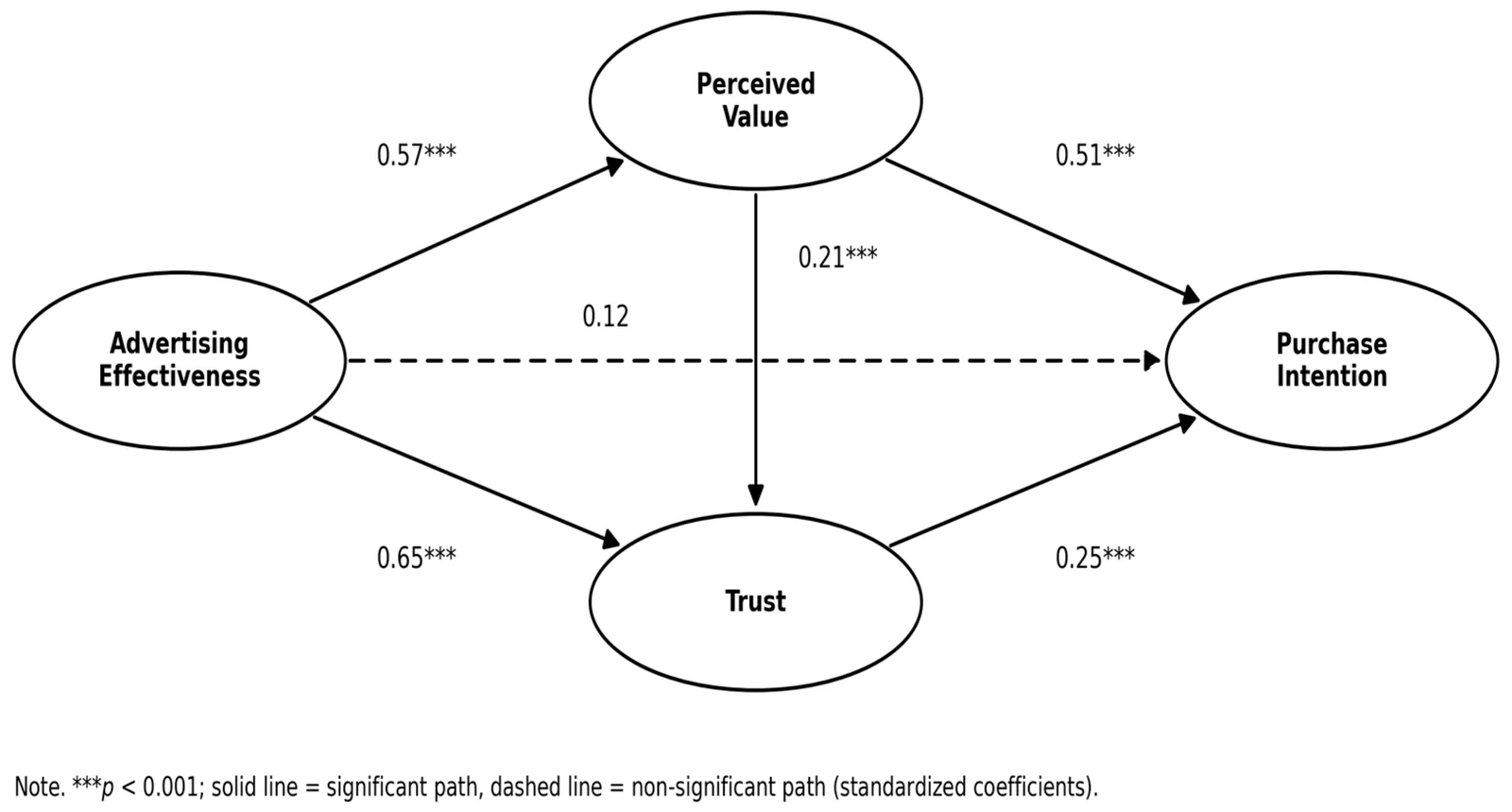
Standardized Path Coefficients of the Research Model.

Structural equation modeling (SEM) was conducted to estimate the path coefficients of the proposed model. The structural model yielded χ^2^(59) = 242.01 (*p* < 0.001), IFI = 0.956, TLI = 0.942, CFI = 0.956, and RMSEA = 0.087; as noted in Section 4.2, the RMSEA lies in the mediocre-but-acceptable band (Browne & Cudeck, 1992) while the incremental indices exceed conventional thresholds. The estimated path coefficients are reported in Table 7 and illustrated in Figure 2.

Advertising effectiveness was significantly positively associated with perceived value (β = 0.57, *p* < 0.001) and trust (β = 0.65, *p* < 0.001). Perceived value, in turn, was positively associated with trust (β = 0.21, *p* < 0.001) and purchase intention (β = 0.51, *p* < 0.001), while trust also showed a significant positive association with purchase intention (β = 0.25, *p* < 0.001). In contrast the direct association between advertising effectiveness and purchase intention was not significant (β= 0.12, *p* = 0.089).

Accordingly, hypotheses H1, H2, H4, H5, and H6 were supported, whereas H3, which posited a direct positive association between advertising effectiveness and purchase intention, was not supported.

Bootstrapping with 2000 resamples was conducted to test the mediating effects of perceived value and trust, and the results are reported in Table 8. The indirect association between advertising effectiveness and purchase intention through perceived value was significant (β = 0.29, *p* < 0.001), as was the indirect association through trust (β = 0.16, *p* < 0.01). In addition, the sequential indirect association through perceived value and trust was also significant (β = 0.03, *p* < 0.01). Although this sequential coefficient is smaller than the single-mediator indirect effects, this is expected rather than anomalous: a three-step path is the product of three structural coefficients and is therefore necessarily attenuated. Its theoretical importance lies in its statistical reliability and its consistency with the scaffolding sequence, not in its absolute magnitude. Accordingly, hypotheses H7, H8, and H9, which proposed the mediating and sequential mediating roles of perceived value and trust, were supported.

### 4.4. Rival Ordering Comparison

Because the sequential ordering of value and trust cannot be settled a priori, we estimated two restricted sequential models in addition to the full structural model: a value-first specification (AE → PV → TR → PI) and a trust-first specification (AE → TR → PV → PI). With identical degrees of freedom (df = 62), the trust-first specification showed better fit (χ^2^ = 301.38, CFI = 0.943, RMSEA = 0.097) than the value-first specification (χ^2^ = 488.54, CFI = 0.898, RMSEA = 0.129), indicating that, when each mechanism is forced to operate alone, trust formation is more directly rooted in advertising effectiveness than in prior value judgments. Critically, however, both restricted models fit worse than the full model (χ^2^(59) = 242.01, CFI = 0.956, RMSEA = 0.087), in which advertising effectiveness is associated with trust both directly (β = 0.65, *p* < 0.001) and indirectly through perceived value (PV → TR: β = 0.21, *p* < 0.001). We therefore do not claim that value categorically precedes trust; rather, the data support a dual-antecedent account in which trust in the AI source is anchored primarily in the perceived effectiveness of the recommendation itself, with consumers’ autonomous value judgments contributing significantly and incrementally to trust formation. It is this incremental value-to-trust pathway—required by the scaffolding framework and empirically supported here—that carries the sequential indirect association of H9.

### 4.5. Moderating Effect Analysis

Multi-group analysis (MGA) was conducted to examine the moderating effects of AI utilization level. Based on the mean value (3.35), respondents were divided into high and low AI utilization groups, and differences in the associations of advertising effectiveness, perceived value, and trust with purchase intention were assessed. Prior to hypothesis testing, measurement invariance was evaluated. Configural invariance was supported, as acceptable model fit was observed for both the low-utilization group (χ^2^(59) = 201.04, IFI = 0.926, TLI = 0.901, CFI = 0.925, RMSEA = 0.097) and the high-utilization group (χ^2^(59) = 106.11, IFI = 0.965, TLI = 0.953, CFI = 0.965, RMSEA = 0.072).

Full measurement invariance, which constrained all factor loadings to be equal across groups, was not supported (Δχ^2^(9) = 21.10, *p* < 0.001). After releasing constraints on selected items with substantial group differences, partial measurement invariance was established (Δχ^2^(6) = 11.65, n.s.).

The results of the structural path comparisons are reported in Table 9. A significant group difference was found for the path from perceived value to purchase intention (Δχ^2^(1) = 5.04, *p* < 0.05), with a stronger positive association observed in the high AI utilization group. In contrast, the associations of advertising effectiveness (Δχ^2^(1) = 0.14, n.s.) and trust (Δχ^2^(1) = 0.20, n.s.) with purchase intention did not differ significantly across groups. Accordingly, hypothesis H10 was supported, whereas hypotheses H11 and H12 were not supported.

### 4.6. Configurational Analysis (fsQCA)

To complement the variable-centered SEM analysis, fsQCA was conducted to identify configurational patterns associated with high purchase intention. Necessity analysis (Table 10) showed that no condition—including advertising effectiveness—reaches the 0.90 consistency benchmark (AE = 0.735, PV = 0.807, TR = 0.731, AIU = 0.805); thus, no single condition is individually necessary. The truth table is reported in Table 11. The conservative solution (Table 12) identified three sufficient configurations. The dominant path combines perceived value with AI utilization (PV·AIU; raw coverage = 0.720, unique coverage = 0.174, consistency = 0.917) and notably does not require advertising effectiveness. The two remaining paths (AE·PV·TR and AE·TR·AIU) each incorporate advertising effectiveness in combination with other conditions. Overall solution coverage was 0.786 with solution consistency of 0.899. Because no condition is individually necessary, the presence of a high-coverage sufficient path that omits advertising effectiveness is an expected expression of equifinality rather than a contradiction: AI advertising operates as one of several substitutable scaffolding inputs, not a singular persuasive driver.

Two findings are particularly noteworthy. First, equifinality is evident: multiple distinct pathways lead to the same outcome, indicating that high purchase intention in experiential tourism cannot be reduced to a single causal recipe. Second the truth table (Table 11) contains six configurations coded as sufficient for high purchase intention (Outcome = 1), that is, rows meeting the consistency (≥0.80) and PRI (≥0.65) thresholds. Of these six, two configurations (AE = 0, PV = 1, TR = 1, AIU = 1, n = 13; and AE = 0, PV = 1, TR = 0, AIU = 1, n = 18) reach the outcome in the absence of advertising effectiveness (AE = 0). Boolean minimization of the six sufficient rows yields the three-path conservative solution reported in Table 12, in which these two AE-absent rows are jointly subsumed by the PV·AIU path—the path with the largest raw (0.720) and unique (0.174) coverage, and one that does not include advertising effectiveness as a condition. Advertising effectiveness is thus dispensable at both levels of the analysis: in two of the six observed sufficient configurations, and in the most prominent path of the minimized solution. This suggests that AI advertising is not an indispensable component but rather one of several combinable inputs—a configurational pattern consistent with the scaffolding framework’s characterization of AI advertising as evaluative support rather than persuasive necessity. 

## 5. Discussion

### 5.1. What AI Advertising Does When Direct Persuasion Fails

The central finding—that AI-targeted advertising is not directly associated with purchase intention for cultural experience tourism (β = 0.12, n.s.)—aligns with and extends two important streams of recent research. Cicek et al. (2024) showed that AI disclosure undermines emotional trust and reduces purchase intention; our finding complements this by demonstrating that even when consumers are exposed to AI-targeted advertising without negative labeling effects, the advertising’s association operates entirely through cognitive mediation rather than direct persuasion. Park and Son (2025) showed that AI recommendations are less effective for hedonic than utilitarian travel goals; our results provide a mechanistic explanation for why: cultural experience tourism, as a hedonic and experiential product category, requires consumers to engage in active value construction that cannot be algorithmically shortcutted.

The specific pattern of full mediation—where the direct path is non-significant while both the single-mediator paths (through perceived value and through trust) and the sequential path (through both) are significant—indicates that AI advertising is associated with a consumer-driven evaluative process rather than directly associated with purchase motivation. This pattern is consistent with the cognitive scaffolding interpretation: AI advertising provides informational cues (about available experiences, their features, their alignment with consumer preferences) that consumers use as raw material for their own value judgments. The “scaffolding” metaphor is apt because, as in Vygotsky’s (1978) original formulation, the structure enables the learner to achieve outcomes they could not reach independently—in this case, identifying and evaluating experiential options across a large information space—without prescribing the outcome itself. Taken together, the empirical results correspond systematically to the three defining properties of cognitive scaffolding theorized a priori. The non-significant direct association between advertising effectiveness and purchase intention (H3 not supported) corresponds to the first property—structure without content: AI advertising provides informational structure but does not directly inject purchase motivation, leaving the consumer to construct value judgments autonomously. The significant sequential mediation through perceived value and trust (H9 supported) corresponds to the second property—zone of proximal development: consumers cannot efficiently evaluate the vast landscape of cultural experience tourism options without AI’s informational support, yet they use the AI-provided cues to autonomously construct value assessments and develop trust, rather than passively receiving pre-formed conclusions. The significant moderation of AI utilization on the perceived value–purchase intention pathway (H10 supported), combined with the absence of moderation on the other pathways (H11, H12 not supported), corresponds to the third property—capability contingency: scaffolding effectiveness varies with the user’s competence, and this variation operates specifically at the point where scaffolded understanding is translated into independent action. This systematic correspondence between a priori theoretical predictions and empirical outcomes supports the interpretation that cognitive scaffolding is not merely a post hoc label applied to a full mediation finding, but a theoretically grounded framework that generates distinctive, falsifiable predictions—each of which was consistent with the data. We emphasize, however, that the non-significant direct path (β = 0.12, *p* = 0.089) is a failure to reject the null rather than affirmative evidence of a zero effect; its 90% confidence interval did not exclude small positive values. What discriminates the scaffolding account from the persuasion account is therefore the joint pattern—significant indirect associations through perceived value and through trust, a significant sequential path, and configurational evidence that advertising effectiveness is neither necessary nor uniquely sufficient—rather than the null direct path alone. This joint interpretation is reinforced by the way our two analytical approaches converge. The SEM and the fsQCA provide convergent, multi-method evidence. The SEM establishes the average mediational structure across respondents, showing that advertising effectiveness operates on purchase intention only indirectly. The fsQCA reinforces and extends this finding at the level of case configurations. Necessity analysis showed that no condition—including advertising effectiveness—is individually necessary for high purchase intention (all consistencies < 0.90), and the conservative solution identified three sufficient paths (Table 12). The dominant path (perceived value combined with AI utilization; PV·AIU) achieves high purchase intention without advertising effectiveness and carries the largest unique coverage, whereas the two remaining paths (AE·PV·TR and AE·TR·AIU) incorporate advertising effectiveness alongside other conditions. We emphasize that this configurational pattern is compatible with the scaffolding framework rather than uniquely confirmatory of it: because no condition is individually necessary, the existence of a high-coverage sufficient path omitting advertising effectiveness is an expected expression of equifinality that reinforces the SEM-based account and is difficult to square with a purely persuasive model, though it does not exclude all alternative explanations. Concretely, the fsQCA adds three things the SEM cannot. First, whereas the SEM estimates an average structure that holds “on average” across respondents, the fsQCA demonstrates equifinality—multiple distinct condition combinations reach the same outcome—which an averaged model masks. Second, the necessity analysis provides a stronger test of the scaffolding claim than mediation alone: showing that advertising effectiveness is not even necessary for high purchase intention is a more demanding result than showing it operates indirectly. Third, the dominant advertising-free path (PV·AIU) identifies the specific consumer profile—high value combined with high AI utilization—for whom advertising is most dispensable, translating the abstract scaffolding prediction into an observable case configuration. The two methods therefore do not merely agree; the fsQCA specifies boundary and heterogeneity conditions that the SEM, by design, cannot reveal.

### 5.2. A Value-Augmented Trust Architecture: Repositioning Value in AI-Mediated Consumer Decisions

A second implication of our findings concerns the ordering of value and trust. This ordering is practically consequential: if trust precedes value assessment, then building consumer trust in AI recommendation systems is the priority. Our rival-model comparison (Section 4.4) indicates that neither pure ordering fully captures the data: trust is anchored primarily in perceived recommendation effectiveness, yet consumers’ value judgments contribute a significant incremental pathway to trust (β = 0.21, *p* < 0.001). The practical priority is therefore twofold—demonstrating recommendation quality remains essential, because consumers who recognize that AI has identified a genuinely valuable experience extend additional trust as a consequence of that recognition, over and above the trust generated by the recommendation experience itself. We stress, however, that because our data are cross-sectional, this value-augmented ordering is offered as consistent with our findings rather than as definitive proof of temporal precedence; establishing the actual sequence in which value and trust form would require longitudinal evidence.

This value-augmented architecture may be specific to the experiential hospitality context examined here, whose value must be actively constructed because it resists pre-consumption verification. For standardized hospitality products (e.g., hotel booking, airline ticketing), where product attributes are directly comparable, trust in the recommendation system may function more directly as a precondition, and persuasion may operate more straightforwardly. The boundary conditions of the value-augmented architecture warrant further investigation across hospitality product types.

This finding also speaks to the emerging framework of Bilgihan et al. (2026), who proposed a dual-target persuasion model for AI-mediated hospitality marketing. While their framework addresses the important question of how persuasion operates when AI agents become parallel decision-makers, our empirical results suggest that for experiential products, the more fundamental question is not whom to persuade (human vs. AI agent) but whether persuasion is the right framework at all. The cognitive scaffolding alternative may better capture AI advertising’s function in contexts where product value resists algorithmic parameterization.

### 5.3. AI Utilization as Cognitive Fluency: Heterogeneous Pathways to Purchase

The moderating role of AI utilization level reveals that consumer heterogeneity in AI-mediated hospitality operates at a specific point in the cognitive process. The perceived value → purchase intention pathway was significantly stronger among high-AI-utilization consumers (β = 0.58 vs. 0.34, *p* < 0.05), while the trust → purchase intention and direct advertising → purchase intention pathways did not differ across groups.

This pattern of selective moderation has three important implications. First, it indicates that AI utilization may function as cognitive fluency in the association between value assessments and behavioral commitments—not as a general amplifier of all AI advertising effects. High-utilization consumers do not perceive more value or trust AI more; the association between recognized value and purchase action is stronger with less friction. This is consistent with Li et al.’s (2024) meta-analytic finding that AI’s service outcomes operate through cognition rather than direct behavioral influence, while specifying where cognitive processing matters most.

Second, the absence of moderation on the trust → purchase intention pathway suggests that trust operates as a universally accessible processing route—a heuristic that works regardless of AI experience level. This finding aligns with Shi et al.’s (2021) distinction between systematic and heuristic processing, but clarifies that the heterogeneity introduced by AI utilization is specific to the systematic (analytical) pathway rather than the heuristic (trust-based) pathway.

Third, the finding extends Seyfi et al.’s (2025) identification of psychological barriers to AI adoption by specifying the cognitive locus of these barriers: they operate not at the evaluation stage (where value is recognized) or the trust stage (where reliability is assessed) but at the action stage (where evaluations are translated into behavioral commitments). This suggests that overcoming AI adoption barriers in experiential hospitality requires not more persuasive advertising or more trustworthy systems, but more effective cognitive support for the value-to-action conversion.

### 5.4. Theoretical Implications

First, this study proposes and provides empirical support for the cognitive scaffolding framework as a lens for understanding AI advertising in experiential hospitality. The full mediation result suggests that AI advertising’s function in this context is to provide informational structure for consumer-driven value construction, not to be directly associated with purchase motivation. This framework extends the understanding of AI’s role beyond the “decision-support” framing common in the tourism technology literature (Gursoy et al., 2023; Lv et al., 2022) by specifying a precise cognitive mechanism: scaffolding operates through value construction, which is linked to trust, which is in turn associated with behavioral commitment. While Li et al.’s (2024) meta-analytic taxonomy classifies what AI features are—sensory, social, or functional—the scaffolding framework specifies what AI advertising does cognitively: it structures the evaluative environment in which consumers construct experiential value. This distinction between feature-level classification and process-level theorization represents a complementary analytical layer for understanding AI’s role in hospitality marketing.

Second, this study provides empirical evidence for a value-augmented trust architecture in AI-mediated experiential hospitality decisions. A formal rival-model comparison showed that trust formation is anchored primarily in perceived recommendation effectiveness—consistent with trust-first and parallel-processing accounts (Gefen et al., 2003; Shi et al., 2021; Bilgihan et al., 2026)—while consumers’ autonomous value judgments contribute a significant incremental pathway to trust that carries the sequential scaffolding mechanism. We propose this architecture as specific to experiential products, whose value resists algorithmic verification, and not as a general account of AI-mediated trust formation; generalization to search or utilitarian goods awaits direct comparative evidence.

Third, the selective moderation of AI utilization—operating at the value-to-action point but not at the evaluation or trust stages—specifies the cognitive locus where individual differences in AI experience matter most. This advances the consumer heterogeneity discussion in AI consumer behavior research (Seyfi et al., 2025; Chi et al., 2022) from identifying that consumers differ in AI responses to identifying where those differences operate in the cognitive process.

Fourth, the full mediation result has implications for how the S–O–R framework is applied in AI-mediated consumer behavior research. The non-significant direct stimulus → response link means that the organism stage—and its internal sequential structure—is the theoretically decisive component. Future applications of S–O–R in AI-mediated consumption contexts should attend carefully to the internal architecture of the organism stage rather than treating it as an undifferentiated mediating mechanism.

Fifth, this study illustrates the value of a hybrid SEM-fsQCA approach for investigating AI advertising effects on consumer behavior. While SEM revealed the net effect structure (sequential mediation, moderation), fsQCA uncovered configurational complexity invisible to variable-centered methods—including the finding that advertising effectiveness is absent from the most prominent sufficient path (PV·AIU) and is not required in every configuration for high purchase intention. This methodological complementarity addresses the growing call in consumer behavior research for multi-method approaches that capture both symmetric and asymmetric causal patterns (Pappas & Woodside, 2021).

Sixth, this study responds to the research agenda outlined by Jain et al. (2024), who identified the cognitive mechanisms of AI-mediated consumer behavior as a critical gap. Our findings specify one such mechanism—cognitive scaffolding—and show that it operates through a value-augmented trust architecture with capability-contingent moderation. This contributes to the broader AI consumer behavior literature by showing that consumers are not passive recipients of algorithmic recommendations but active constructors of experiential value, a finding that speaks to Trang’s (2025) concerns about AI-induced consumer dependency by demonstrating that, at least for experiential products, consumers retain evaluative autonomy even within AI-mediated environments.

### 5.5. Managerial Implications

First, Redesign AI advertising metrics for experiential products. The finding that AI advertising is not directly associated with purchase intention suggests that conventional conversion metrics (click-through rates, immediate booking rates) may fundamentally misrepresent AI advertising’s contribution for experiential products. Marketers of experiential products should adopt value-communication metrics: does the advertising help consumers understand why a particular cultural experience aligns with their preferences? Does it enhance perceived benefit-to-cost assessment? These intermediate outcomes, not direct conversion, are the appropriate performance indicators.

Second, implement a “demonstrate value first” trust strategy. Rather than investing in trust signals (security badges, social proof counts, “trusted by millions”), hospitality platforms should focus on ensuring that AI recommendations consistently identify experiences that consumers subsequently evaluate as valuable. The empirical evidence is consistent with a value-augmented account: trust is anchored primarily in perceived recommendation effectiveness, while value judgments contribute a significant incremental pathway to trust. Platform investment should prioritize recommendation relevance and value communication over trust declarations.

Third, segment by AI utilization for interface design, not message content. The moderating effect of AI utilization operates at the value-to-action conversion point, not at the evaluation stage. This means high- and low-utilization consumers can receive the same AI-generated recommendations, but should encounter different interface designs for the purchase conversion stage. High-utilization consumers benefit from streamlined, data-rich booking interfaces with algorithmic transparency. Lower-utilization consumers benefit from guided conversion pathways: curated comparisons, editorial recommendations (“experiences like this are popular with first-time visitors”), and step-by-step booking support that reduces the cognitive friction of translating AI-mediated evaluations into action.

Fourth, for cultural experience tourism specifically, AI advertising should incorporate experiential previews that enhance perceived value by reducing the algorithmic representation gap: immersive visual content, participant narratives organized by traveler type, and multi-sensory cues that complement algorithmic recommendation with experiential indicators. The goal is not to make AI more persuasive but to give consumers richer material for their own value construction.

These principles cohere into a single design philosophy for AI-mediated experiential marketing. For recommendation systems, foreground value-diagnostic information—why a given experience matches the consumer’s preferences—rather than persuasive appeals. For personalization, calibrate support to the consumer’s inferred competence and allow it to fade as competence grows, mirroring the scaffolding principle rather than applying uniform persuasive pressure. For creative design, emphasize evaluability and the preservation of consumer autonomy over direct persuasion. Across all three, the unifying aim is to support, not override, consumer value construction.

### 5.6. Limitations and Future Research

This study has several limitations that suggest directions for future research. First, the cross-sectional design limits causal inference; longitudinal research tracking consumers from AI advertising exposure through booking and post-experience evaluation would illuminate how the scaffolding mechanism evolves over time. Second, all constructs were measured from a single source; although the confirmatory single-factor comparison argues against a purely method-driven covariance structure, common method variance cannot be entirely excluded. This concern is compounded by the sample itself: reliance on an online panel—together with screening for prior exposure to AI-mediated advertising—may bias the sample toward digitally engaged, AI-receptive consumers, limiting generalization to the broader population. Third, the three defining properties of scaffolding—contingency, fading, and transfer of responsibility—were not directly measured, so our scaffolding interpretation remains inferential; developing construct-level operationalizations of these properties is therefore a priority for future work. A related interpretive caution concerns effect size: while the sequential indirect association through perceived value and trust is statistically significant, its magnitude is small, and its practical significance should be weighed accordingly. Two measurement-related cautions also warrant emphasis. First, although advertising effectiveness and trust satisfy discriminant-validity criteria, their elevated correlation reflects a conceptual proximity that should be borne in mind when interpreting their separate roles; future work may benefit from refined measures that more sharply distinguish evaluation of the advertisement from reliance on the AI source. Second, our operationalization of AI utilization as a proxy for consumer capability is imperfect: a single behavioral-frequency measure cannot fully separate capability from adjacent constructs such as familiarity, technological readiness, or general attitude toward AI, and this interpretation should therefore be treated as provisional rather than settled.

Beyond these design considerations, the findings are bounded in scope. Fourth, the value-augmented architecture is likely specific to experiential products whose value resists pre-consumption verification; it may weaken for utilitarian tourism services or standardized accommodation, where quality is more objectively verifiable and persuasion may operate more directly. Future research should test whether the mechanism extends to other experiential contexts—such as wellness services, live entertainment, or luxury retail—and whether it varies across experience types, for instance whether spiritually intensive experiences (temple stay, meditation) exhibit stronger full mediation than culturally accessible ones (traditional food, craft workshops). Fifth, the study examined Korean consumers, one of the world’s most digitally advanced and AI-familiar populations; because trust in AI and attitudes toward tourism vary cross-nationally along dimensions such as uncertainty avoidance and institutional trust, both the strength and the ordering of the observed associations may differ elsewhere. Cross-cultural replication—following, for example, the comparative framework of Seyfi et al. (2025)—would be especially valuable for testing whether the value-augmented architecture is contingent on baseline familiarity with algorithmic systems. Finally, the fsQCA calibration thresholds followed established practice (Ragin, 2008), but alternative specifications could yield different configurational solutions; future work should include sensitivity analyses to confirm robustness.

## Figures and Tables

**Table 1:** Demographic and Tourism-Related Characteristics of Respondents (*N* = 412).

Variable		*n*	%
Gender	Male	203	49.3
	Female	209	50.7
Age	20s or younger	86	20.9
	30s	68	16.5
	40s	135	32.8
	50s	111	26.9
	60s or older	12	2.9
Education	High school or lower	146	35.4
	Associate degree	65	15.8
	Bachelor’s degree	173	42.0
	Graduate degree	28	6.8
Occupation	Sales	36	8.7
	Office/Administrative	163	39.6
	Professional	29	7.0
	Service/Sales	19	4.6
	Self-employed	67	16.3
	Unemployed	22	5.3
	Student	40	9.7
	Others	36	8.7
Monthly Personal Income	Less than KRW 2 million	38	9.2
	KRW 2–3 million	148	35.9
	KRW 3–4 million	78	18.9
	KRW 4–5 million	46	11.2
	KRW 5 million or more	102	24.8
Region of Residence	Non-metropolitan area	210	51.0
	Metropolitan area	202	49.0
Travel Frequency (Past Year)	Once	18	4.4
	Twice	219	53.2
	3–4 times	99	24.0
	5 times or more	76	18.4
Primary Booking Channel	OTA	290	70.4
	Official website	60	14.6
	Travel agency	43	10.4
	SNS/Links	19	4.6

**Table 2:** Validity and Reliability of the Measurement Scales.

Factor	Item	Factor Loading	Eigen Value	Variance (%)	Cronbach’s α
Perceived Value	Perceived Value2	0.848	2.78	17.40	0.94
	Perceived Value1	0.821			
	Perceived Value3	0.809			
Advertising Effectiveness	Advertising Effectiveness2	0.869	2.72	17.01	0.87
	Advertising Effectiveness1	0.838			
	Advertising Effectiveness4	0.620			
	Advertising Effectiveness3	0.532			
Trust	Trust1	0.808	2.58	16.10	0.86
	Trust2	0.807			
	Trust3	0.656			
Purchase Intention	Purchase Intention1	0.810	2.49	15.54	0.90
	Purchase Intention2	0.749			
	Purchase Intention3	0.736			
AI Utilization	AI Utilization1	0.791	2.43	15.18	0.88
	AI Utilization2	0.780			
	AI Utilization3	0.718			
Total				81.23	-
KMO		0.923			
Bartlett’s Test		χ^2^(120) = 5275.75(0.000)			

**Table 3:** Descriptive Statistics of Main Variables (*N* = 412).

Variable	M	SD	Skewness	Kurtosis
Advertising Effectiveness	3.67	0.76	−0.20	−0.37
Perceived Value	3.46	0.72	0.16	−0.29
Trust	3.59	0.74	0.07	−0.39
AI Utilization	3.35	0.69	0.22	0.29
Purchase Intention	3.15	0.85	−0.15	−0.17

**Table 4:** Correlations Between Key Variables (*N* = 412): Correlations are Pearson coefficients computed on raw composite (item-mean) scores.

Variable	1	2	3	4	5
1. Advertising Effectiveness	1				
2. Perceived Value	0.53 ***	1			
3. Trust	0.72 ***	0.54 ***	1		
4. AI Utilization	0.54 ***	0.68 ***	0.55 ***	1	
5. Purchase Intention	0.54 ***	0.65 ***	0.57 ***	0.69 ***	1

*** *p* < 0.001.

**Table 5:** Convergent Validity: Factor Loadings, AVE, and CR.

Latent Variable	Measurement Variable	*β*	AVE	CR
Advertising Effectiveness	Advertising Effectiveness1	0.85	0.62	0.87
	Advertising Effectiveness2	0.82		
	Advertising Effectiveness3	0.76		
	Advertising Effectiveness4	0.72		
Perceived Value	Perceived Value1	0.90	0.84	0.94
	Perceived Value2	0.94		
	Perceived Value3	0.90		
Trust	Trust1	0.85	0.69	0.87
	Trust2	0.90		
	Trust3	0.73		
Purchase Intention	Purchase Intention1	0.81	0.75	0.90
	Purchase Intention2	0.87		
	Purchase Intention3	0.91		

**Table 6:** Discriminant Validity (Square Root of AVE on Diagonal). Off-diagonal entries are latent construct correlations estimated in the CFA; diagonal entries are the square roots of the AVE. Latent correlations exceed the raw-score correlations in Table 4 because they are disattenuated for measurement error; the CFA-based values are authoritative for discriminant validity assessment.

Variable	1	2	3	4
1. Advertising Effectiveness	(0.81)			
2. Perceived Value	0.57	(0.92)		
3. Trust	0.77	0.58	(0.83)	
4. Purchase Intention	0.59	0.72	0.63	(0.88)

**Table 7:** Path Coefficients in the Structural Model.

Path	B	SE	*β*	*t*
Advertising Effectiveness → Perceived Value	0.60	0.06	0.57	10.34 ***
Advertising Effectiveness → Trust	0.57	0.06	0.65	9.76 ***
Advertising Effectiveness → Purchase Intention	0.15	0.09	0.12	1.60
Perceived Value → Trust	0.18	0.04	0.21	4.28 ***
Perceived Value → Purchase Intention	0.61	0.07	0.51	9.49 ***
Trust → Purchase Intention	0.35	0.11	0.25	3.35 ***

*** *p* < 0.001.

**Table 8:** Results of Mediating Effects Analysis.

Path	B	SE	*β*
Advertising Effectiveness → Perceived Value → Purchase Intention	0.37	0.05	0.29 ***
Advertising Effectiveness → Trust → Purchase Intention	0.20	0.07	0.16 **
Advertising Effectiveness → Perceived Value → Trust → Purchase Intention	0.04	0.02	0.03 **

** *p* < 0.01, *** *p* < 0.001.

**Table 9:** Results of Moderating Effects Analysis.

Path	*β*		Δχ^2^
	Low (*n* = 258)	High (*n* = 154)	
Perceived Value → Purchase Intention	0.34 ***	0.58 ***	5.04 *
Advertising Effectiveness → Purchase Intention	0.08	0.13	0.14
Trust → Purchase Intention	0.29 **	0.18	0.20

* *p* < 0.05, ** *p* < 0.01, *** *p* < 0.001.

**Table 10:** Necessity Analysis for High Purchase Intention (*N* = 412).

Condition	Consistency (*inclN*)	Relevance (*RoN*)	Coverage (*covN*)
AE	0.735	0.872	0.836
PV	0.807	0.865	0.852
TR	0.731	0.883	0.848
AIU	0.805	0.885	0.870

Note. inclN = consistency of necessity; RoN = relevance of necessity; covN = coverage of necessity. A condition is considered necessary when inclN ≥ 0.90 (Schneider & Wagemann, 2012). No condition reaches this threshold, indicating that no single condition is individually necessary for high purchase intention. RoN values well above 0.50 confirm that these necessity assessments are non-trivial.

**Table 11:** Truth Table for High Purchase Intention.

AE	PV	TR	AIU	*n*	Consistency	PRI	Outcome
1	1	1	1	73	0.956	0.907	1
0	1	1	1	13	0.947	0.781	1
1	1	0	1	16	0.943	0.783	1
1	0	1	1	10	0.937	0.686	1
1	1	1	0	20	0.933	0.699	1
0	1	0	1	18	0.909	0.653	1
0	1	1	0	9	0.919	0.580	0
1	1	0	0	7	0.918	0.554	0
1	0	1	0	21	0.887	0.448	0
0	0	1	0	22	0.886	0.428	0
1	0	0	0	14	0.871	0.305	0
0	0	0	1	17	0.871	0.417	0
0	1	0	0	34	0.849	0.422	0
0	0	0	0	131	0.655	0.144	0
0	1	1	1	3	0.937	0.630	?
1	0	0	1	4	0.931	0.578	?

Note. Frequency cutoff = 5; raw consistency ≥ 0.80; PRI ≥ 0.65. Configurations 15–16 (*n* = 7 total) fell below the frequency cutoff and were treated as logical remainders (?). Total *N* = 412. 1 = presence (membership > 0.5); 0 = absence.

**Table 12:** Sufficient Configurations for High Purchase Intention (Conservative Solution).

Configuration	Raw Coverage	Unique Coverage	Consistency
PV × AIU	0.720	0.174	0.917
AE × PV × TR	0.577	0.031	0.937
AE × TR × AIU	0.581	0.035	0.942
Solution	0.786	-	0.899

Note. Solution coverage = 0.786; solution consistency = 0.899. Consistent with necessity results, advertising effectiveness (AE) is absent from the dominant path (PV × AIU), which alone accounts for the largest unique coverage.

## Data Availability

The data presented in this study are openly available via Google Drive at the following link: https://docs.google.com/spreadsheets/d/1wzTqHAyFQQxO7n3qzQDSLhlcyRNYwdLQpQHg2qTNK6U/edit?usp=sharing (accessed on 1 July 2026).

## Appendix A. Measurement Items

All items were measured on a 5-point Likert scale (1 = strongly disagree to 5 = strongly agree). Items were originally administered in Korean and translated into English for reporting purposes using back-translation procedures.

Advertising Effectiveness (adapted from MacKenzie & Lutz, 1989; Ducoffe, 1996).

Code	Item
AE1	The AI-recommended cultural experience tourism advertisement provided useful information.
AE2	The AI-recommended cultural experience tourism advertisement was relevant to my interests.
AE3	The AI-recommended cultural experience tourism advertisement was credible.
AE4	Overall, I found the AI-recommended cultural experience tourism advertisement to be effective.
Perceived Value (adapted from Zeithaml, 1988)	

Code	Item
PV1	The cultural experience tourism recommended by AI offers good value relative to the cost.
PV2	The expected benefits of participating in AI-recommended cultural experience tourism outweigh the anticipated costs.
PV3	Overall, AI-recommended cultural experience tourism provides high value to me.
Trust (adapted from Morgan & Hunt, 1994; Gefen et al., 2003)	

Code	Item
TR1	I trust that the AI recommendation system provides reliable information about cultural experience tourism.
TR2	I believe the AI recommendation system is competent in suggesting cultural experiences that suit me.
TR3	I feel confident in the cultural experience tourism recommendations provided by AI.
Purchase Intention (adapted from Dodds et al., 1991; Ajzen, 1991)	

Code	Item
PI1	I intend to participate in the cultural experience tourism recommended by AI.
PI2	I am likely to book a cultural experience tourism program recommended by AI.
PI3	I am willing to pay to participate in AI-recommended cultural experience tourism.
AI Utilization Level (adapted from Compeau & Higgins, 1995; Venkatesh et al., 2003)	

Code	Item
AIU1	I frequently use AI-based tools (e.g., chatbots, recommendation systems) for information search.
AIU2	I am experienced in using AI technology to support my decision-making.
AIU3	I actively utilize AI-based services in my daily life.
